# 3D Bioprinting of Macroporous Materials Based on Entangled Hydrogel Microstrands

**DOI:** 10.1002/advs.202001419

**Published:** 2020-07-19

**Authors:** Benjamin Kessel, Mihyun Lee, Angela Bonato, Yann Tinguely, Enrico Tosoratti, Marcy Zenobi‐Wong

**Affiliations:** ^1^ Department of Health Sciences and Technology HPL J 22 Otto‐Stern‐Weg 7 Zürich 8093 Switzerland

**Keywords:** bioinks, cartilage, extrusion bioprinting, microgels, tissue engineering

## Abstract

Hydrogels are excellent mimetics of mammalian extracellular matrices and have found widespread use in tissue engineering. Nanoporosity of monolithic bulk hydrogels, however, limits mass transport of key biomolecules. Microgels used in 3D bioprinting achieve both custom shape and vastly improved permissivity to an array of cell functions, however spherical‐microbead‐based bioinks are challenging to upscale, are inherently isotropic, and require secondary crosslinking. Here, bioinks based on high‐aspect‐ratio hydrogel microstrands are introduced to overcome these limitations. Pre‐crosslinked, bulk hydrogels are deconstructed into microstrands by sizing through a grid with apertures of 40–100 µm. The microstrands are moldable and form a porous, entangled structure, stable in aqueous medium without further crosslinking. Entangled microstrands have rheological properties characteristic of excellent bioinks for extrusion bioprinting. Furthermore, individual microstrands align during extrusion and facilitate the alignment of myotubes. Cells can be placed either inside or outside the hydrogel phase with >90% viability. Chondrocytes co‐printed with the microstrands deposit abundant extracellular matrix, resulting in a modulus increase from 2.7 to 780.2 kPa after 6 weeks of culture. This powerful approach to deconstruct bulk hydrogels into advanced bioinks is both scalable and versatile, representing an important toolbox for 3D bioprinting of architected hydrogels.

## Graphical abstract

Bulk gels are deconstructed into hydrogel microstrands to form structured materials with interesting properties for tissue engineering. Preparation of this macroporous material is facile, cell‐friendly, and material independent. Individual microstrands align through extrusion and facilitate orientation in biological structures. Entangled microstrands are an excellent bioink for extrusion 3D bioprinting and can be utilized as a platform for tissue engineering.

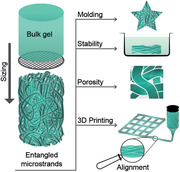

## 1 Introduction

Microextrusion 3D bioprinting has recently been used to create functional analogues of many tissues including myocardium, skeletal muscle, liver, skin, bone, and cartilage.^[^ ^1^ ^]^ Importantly, in vivo evaluation of bioprinted constructs have shown key advantages compared to non‐printed, control samples made from bulk materials.^[^ ^2^ ^]^ The field has profited from a flurry of recent hardware and material advances, however existing bioinks still have critical limitations. One major challenge is associated to the control of their flow properties which permits reproducible and accurate bioprinting with high biocompatibility. Commonly, bioinks are prepared from a solution of hydrogel precursors whose rheological properties have been tuned so the bioink flows as a continuous strand when extruded. To create a 3D object, strands are collected and stacked in a layer‐by‐layer fashion. The challenge of extrusion bioprinting, referred to as a “race against instabilities”, is to prevent flow of extruded material to preserve the fidelity of the print until it can be stabilized by crosslinking.^[^ ^3^ ^]^

To address the flow challenges of common bioinks, the field has resorted to a number of approaches. Increasing the polymer content of bioinks enhances printability, but the resulting densely crosslinked networked can affect cell viability and spreading.^[^ ^4^ ^]^ Methods to print low content bioinks include: 1) the addition of flow modifying fillers or additives, 2) printing within a sacrificial support material, 3) partially crosslinking the bioink before or during extrusion to increase yield stress, 4) templating using a rapidly crosslinkable material, often alginate, and 5) sequential crosslinking of individual layers directly after deposition.^[^ ^5^ ^]^ In general, stability of structures during printing is challenging given the high water content of most bioinks and the tendency of heavy and tall structures to sag under their own weight.^[^ ^6^ ^]^ To make matters more challenging, many approaches, which improve printability, have a detrimental effect on the biological properties of the material.^[^ ^7^ ^]^ There is a pressing need for advanced bioinks which combine both excellent printing and bioactive properties.^[^ ^8^ ^]^

A recent development to tackle this problem are dynamic bioinks.^[^ ^9^ ^]^ Supramolecular bioinks, such as those based on guest–host interactions, or dynamic covalent bioinks based on hydrazone or boronate crosslinking, have been shown to exhibit shear thinning, shear recovery, and self‐healing properties while still being cytocompatible.^[^ ^10^ ^]^ Challenges of these dynamic systems are the transient nature of bonds which can lead to erosion of the hydrogel over time and limited improvement of their porosities, which are still comparable to other bulk hydrogels.^[^ ^9^ ^]^

Alternatively, microgel bioinks have been proposed as a promising strategy, which could fulfill both requirements.^[^ ^11^ ^]^ Compared to liquid bioinks, microgel bioinks have the advantage that they have little intrinsic flow behavior. Such bioinks have inherent shear thinning and shear recovery properties based on weak particle‐particle interactions which are disrupted at high shear stresses during extrusion in the nozzle and reformed during the post‐print phase.^[^ ^5c^ ^]^ When the microgels are “jammed” into a close‐packed state, they have moderate printing properties.^[^ ^12^ ^]^ Simultaneously, due to the inherent void space between the microgels in a close‐packed state, cells within these materials have enhanced viability, spreading and migration compared to bulk monolithic materials.^[^ ^13^ ^]^

Current approaches to make spherical microgels for extrusion bioprinting and other biomedical applications include spraying, microfluidic, emulsion, and stereolithography.^[^ ^2^ ^]^ Despite many advantages, such approaches are still associated with important limitations, including poor scalability, the need for oils, additives, and/or restriction to low viscous polymer solutions. Another strong limitation of these approaches relates to the sphericity of the microgels themselves, as the close‐packed lattice limits interaction between individual spheres and precludes any anisotropy of the printed material. Consequently, current microgel bioinks require a secondary crosslinking to be stable in aqueous media and do not provide important guidance cues to align cells of anisotropic tissues like muscle and tendon.

The use of high‐aspect‐ratio microgels has long been explored in tissue engineering. In fact, cell‐laden hydrogel microfibers have been used as a potent building block.^[^ ^14^ ^]^ In addition, the use of elongated microgels has shown to be useful in guidance of neurons.^[^ ^15^ ^]^ Recently, scaffolds prepared from wet‐spun micro‐ribbons showed the possible advantages of anisotropic gels in cartilage engineering.^[^ ^16^ ^]^ Suspensions of very‐high‐aspect‐ratio fibers have been achieved with microfluidics that enabled the investigation of flow properties of elongated microgels and preparation of hydrogels through entanglement of microfibers.^[^ ^17^ ^]^ However, a facile and cell friendly method to produce large volumes of high‐aspect‐ratio microgels has still not been achieved. This paper presents a new class of microgels, termed entangled microstrands, which overcomes many of the disadvantages of spherical microgel materials and demonstrates the first use of elongated microgels in 3D bioprinting.

**Figure 1:**
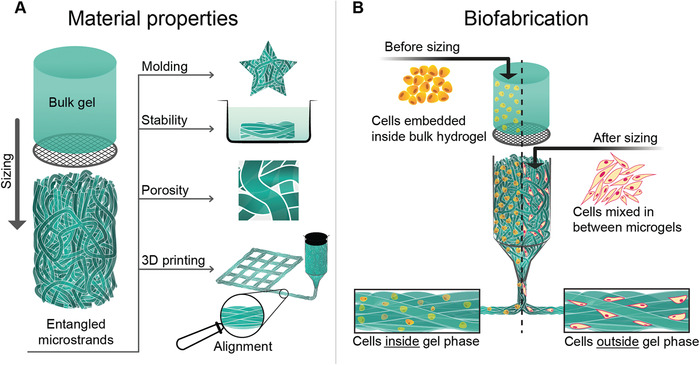
Graphical abstract: A) Bulk hydrogel is mechanically extruded through a grid to deconstruct it into microstrands. In this process, microstrands randomly entangle within each other and form entangled microstrands—a stable material with properties relevant for tissue engineering: moldability, stability in aqueous solutions, porosity, printability, and alignment of microstrands by extrusion. B) A bioink can be created by embedding cells in bulk hydrogel before sizing that results in a spatial deposition of cells inside the gel phase. Alternatively, cells can be mixed in between already prepared entangled microstrands, so cells occupy the space outside the gel phase.

Entangled microstrands were created by pressing a bulk hydrogel through a grid with micron‐sized apertures to deconstruct the hydrogel into individual microstrands (**Figure** **1**A). The production is fast, requires no specialized equipment, and can be used with a wide range of hydrogels of arbitrary polymer content, composition, crosslinking chemistry, and crosslinking density. Due to the simplicity of the production method, it can be upscaled to liter volumes. The void space between strands forms an interconnected porous network and entangled microstrands exhibit long term stability in aqueous solutions, even without secondary crosslinking. The material is moldable and exhibits all relevant rheological properties for 3D bioprinting. Furthermore, cells can be included in the bulk gel or added to the individual microstrands during printing (Figure 1B). High‐fidelity 3D printing could be achieved with a wide range of materials, even with polymer concentrations that typically require the use of support baths or additives to be printable. Finally, the power of this approach for tissue engineering is demonstrated by 3D bioprinting chondrocytes in hyaluronan hydrogel microstrands, showing the in vitro maturation of these constructs into a tissue with mechanical properties approaching that of native cartilage.

## 2 Results

### 2.1 Entangled Microstrands Are Moldable, Stable in Water and Macroporous

**Figure 2:**
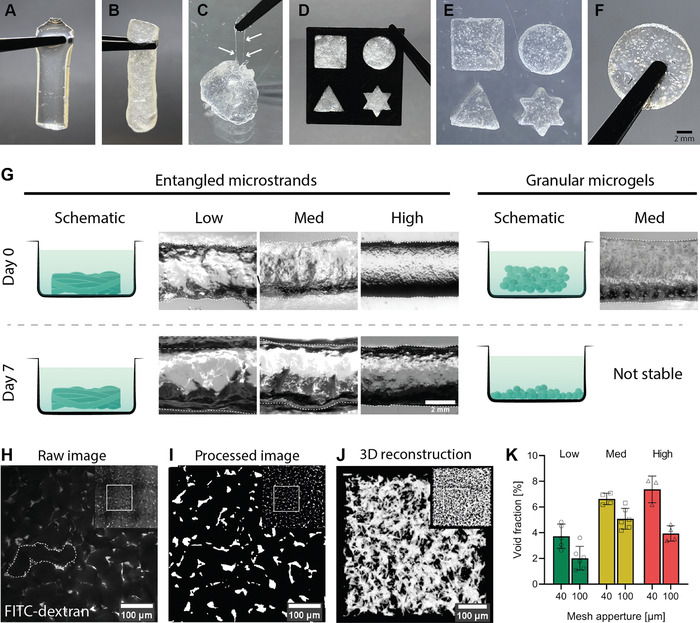
Stability and macroporosity of entangled microstrands: A) Crosslinked bulk HA‐MA hydrogel. B) Entangled microstrands prepared from such bulk hydrogel. C) When entangled microstrands are extended, single microstrands become visible (arrows). D) Entangled microstrands are moldable. E) Secondarily crosslinked custom shapes created by casting. F) Secondary crosslinking tightly anneals microstrands. G) Entangled microstrands show long‐term stability in aqueous solution even without secondary crosslinking, while granular microgels lose cohesion and disintegrate (*n* = 3). H) Multiphoton image of entangled microstrands submerged in FITC‐dextran. I) The same image after processing with a thresholding algorithm. J) 3D reconstruction of the porous network. K) Void fraction significantly varies with crosslinking density (*p* < 0.001) as well as mesh size (*p* < 0.001). Data are presented as individual values as well as mean ± SD (a minimum of three samples per condition were analyzed). Significance was obtained from two‐way ANOVA.

Here, we report a robust and versatile method for preparing “entangled” microstrands using hyaluronan‐methacrylate (HA‐MA) as a model system. Bulk HA‐MA hydrogels were mechanically pressed through a sieve with pores ranging from 40 to 100 µm. This process deconstructed the gel into microstrands, which randomly entangled within each other and made up a structured material consisting exclusively of high‐aspect‐ratio hydrogels. Passing a 2% w/v bulk HA‐MA (degree of substitution 0.28, UV‐A exposure; Figure S1, Supporting Information) through a 40 µm sieve, resulted in a visibly opaque, macroporous material permeable to dyes (**Figure** **2**A,B and Movie S1, Supporting Information). When entangled microstrands were probed with forceps, single microstrands could be visualized (Figure 2C). Entangled microstrands were also deformable and moldable (Figure 2D). Secondary crosslinking of entangled microstrands (UV‐A exposure) created a rigid, macroporous structure which could be handled with forceps (Figure 2E,F).

To investigate the tunability of entangled microstrands for 3D printing, a range of different HA‐MA bulk gels were prepared. Crosslinking of HA‐MA gels was terminated at three different time points, to create bulk hydrogels with a low (Low), medium (Med), and high (High) degrees of crosslinking (Figure S2A, Supporting Information). These hydrogels had significantly different mechanical properties: storage moduli (*n* = 9, *p* < 0.001), compression moduli (*n* = 5, *p* < 0.001), maximum elongation until rupture (*n* = 3, *p* < 0.001), and swelling behavior (*n* = 3, *p* < 0.001; Table S1 and Figure S2B–E, Supporting Information). These three distinctively different bulk hydrogels were then sized through nylon meshes of two different apertures (40 and 100 µm) to create a total of six different variants of entangled microstrands.

Entanglement of microstrands allowed for long‐term cohesion in aqueous medium, when compared to repeatedly sized, granular microgels. To compare stability, entangled microstrands and granular microgels were submerged in PBS for up to 7 days at 37 °C with constant agitation (Figure 2G). All six entangled microstrand materials (40 and 100 µm, Low, Med, High crosslinking) were stable for the entire period without secondary crosslinking, while all granular microgel materials based on the same hydrogels dissociated within 1 h of incubation (*n* = 3).

Porosity is a critical property of materials employed in tissue engineering as this parameter strongly influences transport of nutrients, gas exchange, and cell activity.^[^ ^18^ ^]^ Pore size is also relevant for blood vessel infiltration as well as cell migration.^[^ ^19^ ^]^ To assess the porosity of HA‐MA entangled microstrands, freshly prepared entangled microstrands were submerged in a fluorescent high‐molecular‐weight dextran dye. Figure 2H shows a multiphoton image of the dye distribution taken within the central region of the structure. The void space and hydrogel strand could be clearly distinguished. The labeled dextran could enter the space between individual microstrands, but due to the high molecular weight, dextran was unable to penetrate the gel phase of the hydrogel. Since the dye was detectable within the central region and diffusion through the hydrogel microstrands was not possible, the pore space in between microstrands deemed to be interconnected. To calculate the void fraction of entangled microstrands, images were thresholded to achieve distinct transitions between microstrands and pores. An acquired image before and after processing can be seen in Figure 2H,I. A 3D reconstruction of the interconnected network can be seen in Figure 2J while a quantification of the void fraction is displayed in Figure 2K. Calculated void fractions ranged from 2.0 ± 0.8% for the Low (100 µm) condition to 7.4 ± 0.9% for High (40 µm). Void fraction significantly varied with crosslinking density (*n* = 3, *p* < 0.001) as well as mesh size (*n* = 3, *p* < 0.001).

### 2.2 Entangled Microstrands Have Shear Thinning/Recovery Properties and Clear Yield Points

**Figure 3:**
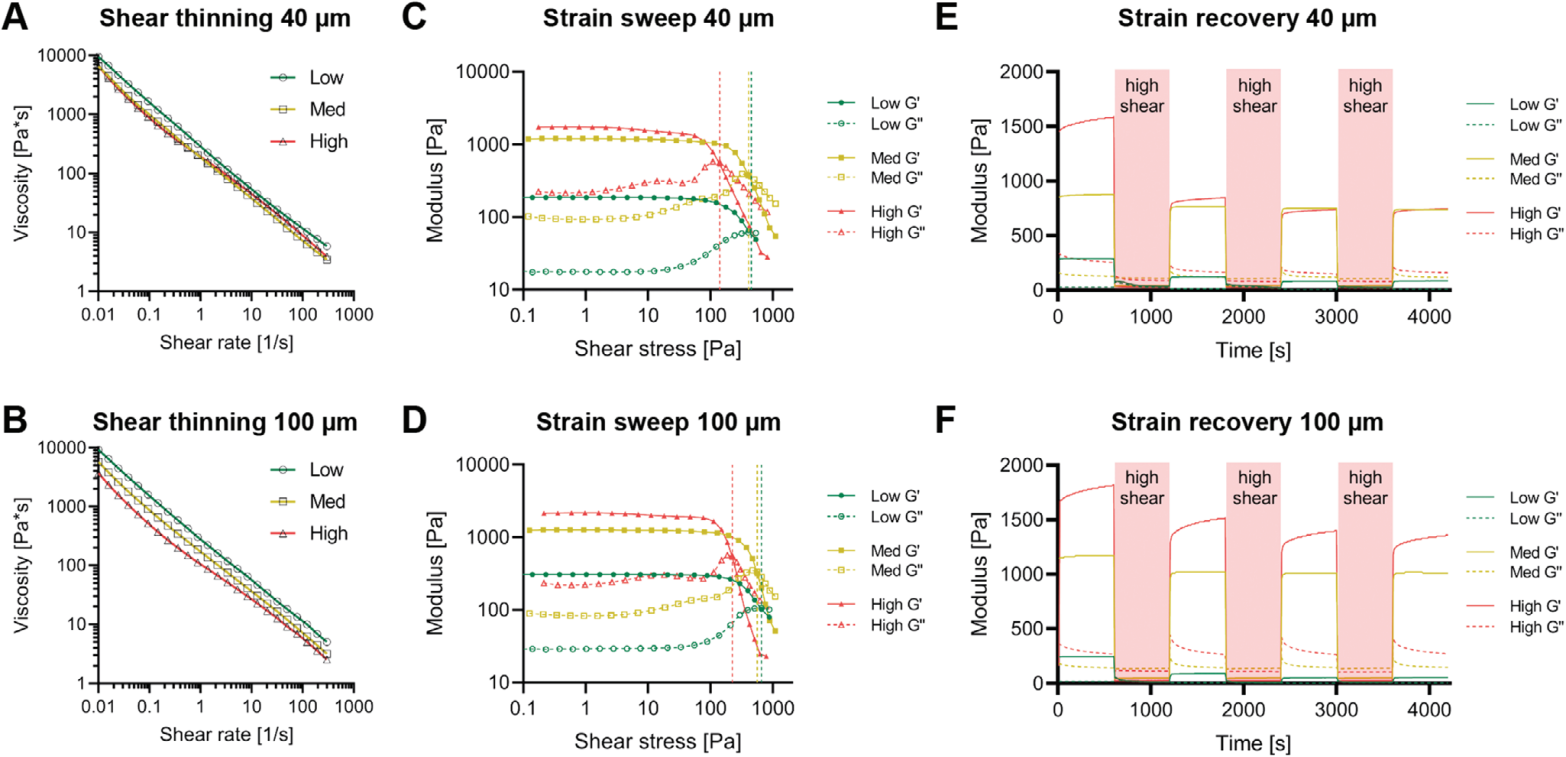
Rheological characterization of HA‐MA entangled microstrands: A,B) Entangled microstrands created by sizing with a grid with aperture size of 40 and 100 µm exhibit shear thinning behavior. C,D) Clear flow points can be determined (Crossover points for 40 µm samples: Low = 450 Pa, Med = 409 Pa, High = 141 Pa; 100 µm samples Low = 659 Pa, Med = 559 Pa, High = 225 Pa). E,F) When subjected to repeated cycles of low and high shear, shear thinning, and shear recovery behavior can be observed for all conditions.

Entangled microstrands exhibited all relevant rheological properties necessary for extrusion 3D bioprinting. All prepared variants of entangled microstrands showed shear thinning behavior (**Figure** **3**A,B). To identify the yield stress necessary to induce flow, different methods can be employed.^[^ ^20^ ^]^ In this study we used the crossover of storage modulus (*G*′) and the loss modulus (*G*′′) to identify the flow point. The required stress to reach crossover was lowest in High samples and highest in Low samples for both mesh apertures (40 µm: Low = 450 Pa, Med = 409 Pa, High = 141 Pa; 100 µm: Low = 659 Pa, Med = 559 Pa, High = 225 Pa, Figure 3C,D). To simulate the printing process, shear recovery tests based on oscillatory strain sweeps with cycles of high and low strain were conducted. At low strains, microstrands exhibited a solid‐like elastic behavior (*G*′ > *G*′′) that rapidly changed into a viscous liquid‐like behavior (*G*′ < *G*′′) when high strains were applied (Figure 3E,F). These transitions are crucial for high‐quality 3D printing as it ensures even material flow during extrusion and shape retention upon deposition on the collector plate.

Shear recovery varied between conditions (Figure S3, Supporting Information). Med samples exhibited nearly optimal shear recovery with 81.8% (40 µm) and 82% (100 µm) recovery after initial shear. This behavior was also similar after subsequent shear when 80.3% (40 µm) and 81.5% (100 µm) of the initial modulus was recovered. High (100 µm) showed slightly less shear recovery with 70.9% (initial) and 65.9% (subsequent) recovery. High (40 µm) recovered to 46.7% of its initial modulus after shear was applied for the first time and 40.8% after subsequent application. In Low samples, even less of the initial modulus was recovered for 40 µm (34.3% and 20.8%) as well as for 100 µm (24.6% and 10.2%) after initial and subsequent shear, respectively. The superior recovery rate makes Med samples the most promising candidate for a bioprinting application when high resolution is required.

### 2.3 Entangled Microstands Have Excellent Printability and Form Anisotropic Structures When Printed

**Figure 4:**
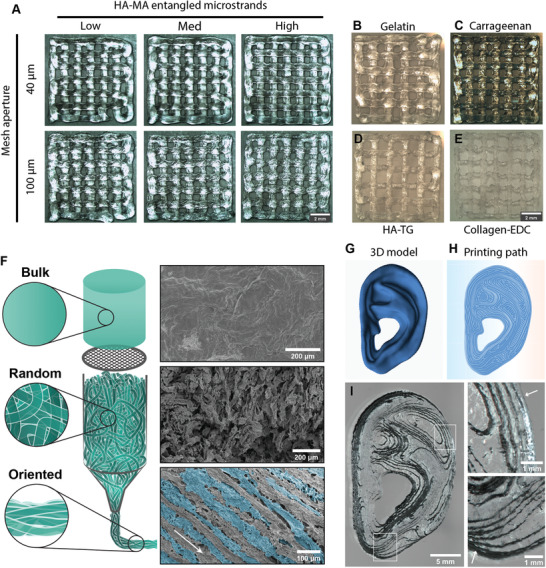
Entangled microstrands are printable and align during extrusion: A) Different HA‐MA entangled microstrands printed in a grid structure. B–E) Entangled microstrands made and printed from gelatin. B) (thermal crosslinking), iota‐carrageenan, C) (ionic crosslinking), (HA‐TG), D) (enzymatic crosslinking), Collagen‐EDC, E) (carbodiimide crosslinking). F) 3D model of a human shaped ear. G) Printing path for 3D printing. H) Ear‐shaped construct 3D‐printed with entangled microstrands made from iota‐carrageenan (arrows point toward sharp transitions between layers). I) Schematic and scanning electron microscopy images of bulk gel, entangled microstrands in random orientation and aligned microstrands after extrusion through a printing nozzle (the arrow indicates the direction of extrusion).

Two‐layered grid structures were printed with entangled microstrands made from HA‐MA (**Figure** **4**A). All six variants were printed with good shape retention and printing resolution. Anomalies arose for lower crosslinked samples when sharp edges were printed. This phenomenon was especially pronounced in Low (40 µm) samples where sharp edges of the model printed with a very rounded appearance and the filament was dragged away during printing. This problem could be explained by the higher mechanical strength of the microstrands compared to that of a typical polymer solution. In typical bioinks, deposited filaments and the reservoir within the printing nozzle are separated as soon as the printing head retracts. In Low microstrand samples, however, microstrands spanned the distance between the printed construct and the printing nozzle. This connection did not rupture right away, but instead the deposited filaments were slightly dragged to the new printing position. Since the more crosslinked samples Med and High were more brittle and ruptured at lower elongation distances, the printing accuracy was higher in these samples. Also taking into account the rheological properties of entangled microstrands, the Med conditions are the most favorable for use as a bioink in extrusion bioprinting. No clear differences could be observed in the rheological properties and printing behavior of the two different aperture sizes (40 and 100 µm). However, aperture size did have a significant impact on void fraction, so this parameter can be chosen according to the desired application to tune macroporosity.

Since all variants of microstrands made from HA‐MA were successfully printed, we investigated the versatility of the approach by using other hydrogel systems commonly used in tissue engineering and 3D culture. The tested systems included gelatin, a thermoresponsive denatured form of collagen together with its photoresponsive derivative, gelatin‐methacrylol (Figure S4, Supporting Information, GelMA), iota‐carrageenan, a highly sulfated polysaccharide that forms ionic crosslinks upon addition of monovalent as well as divalent cations, and enzymatically or chemically crosslinked hyaluronan hydrogels (HA‐TG, HA‐DVS).^[^ ^21^ ^]^ Finally, collagen, a material prevalent in tissue engineering because of its abundance in the extracellular matrix of many tissues, was crosslinked with carbodiimide chemistry.^[^ ^21e^ ^]^ Although derived from a wide range of materials and crosslinking methods, all bulk hydrogels were successfully sized and the entangled microstrands could be 3D printed according to the grid model used for the HA‐MA microstrands (Figure 4B–E and Figure S5, Supporting Information).

In a next step, we explored the potential of microstrands by bioprinting large, complex constructs. A biologically relevant structure was printed with entangled microstrands made from bulk iota‐carrageenan (ear, Figure 4F–H). This 3D model represents a human ear printed at 50% size and demonstrates the power of this approach to create macro‐sized scaffolds (28 × 14 × 7 mm). Printed structures were stable and no flow of the bioink was observed, even after 15 layers were stacked in *z*‐direction. Moreover, individual layers and deposited filaments remained visible and clearly reproduced the printing path created by the slicing software (Figure 4H).

Anisotropy in tissues like muscle, tendon, or nerves is difficult to replicate with conventional tissue engineering approaches. Several studies have shown the possibility to align (nano‐)fibers and anisotropic particles in the direction of flow.^[^ ^22^ ^]^ Extruded microstrands showed alignment in the 3D printing direction, as observed after HA‐MA microstrands were extruded through a 410 µm conical printing nozzle and imaged with scanning electron microscopy (Figure 4I). Clear differences between bulk gel and microstrands (pre‐ and post‐printed) were apparent. Bulk hydrogels had an even surface, whereas microstrands before printing were randomly entangled within each other and had no clear orientation. Extrusion through a nozzle oriented the microstrands and post‐printing, microstrands were aligned in the direction of printing.

### 2.4 Cellular Entangled Microstrands Lead to Rapid Tissue Maturation

To explore the potential for 3D bioprinting with entangled microstrands, two possible cell delivery approaches were investigated (Figure 1B). First, cells were embedded inside the bulk hydrogel before the hydrogel was sized into microstrands to create cell‐laden microstrands (Inside). Alternatively, entangled microstrands were mixed with a cell suspension, leaving cells to occupy the void space between microstrands (Outside).

**Figure 5:**
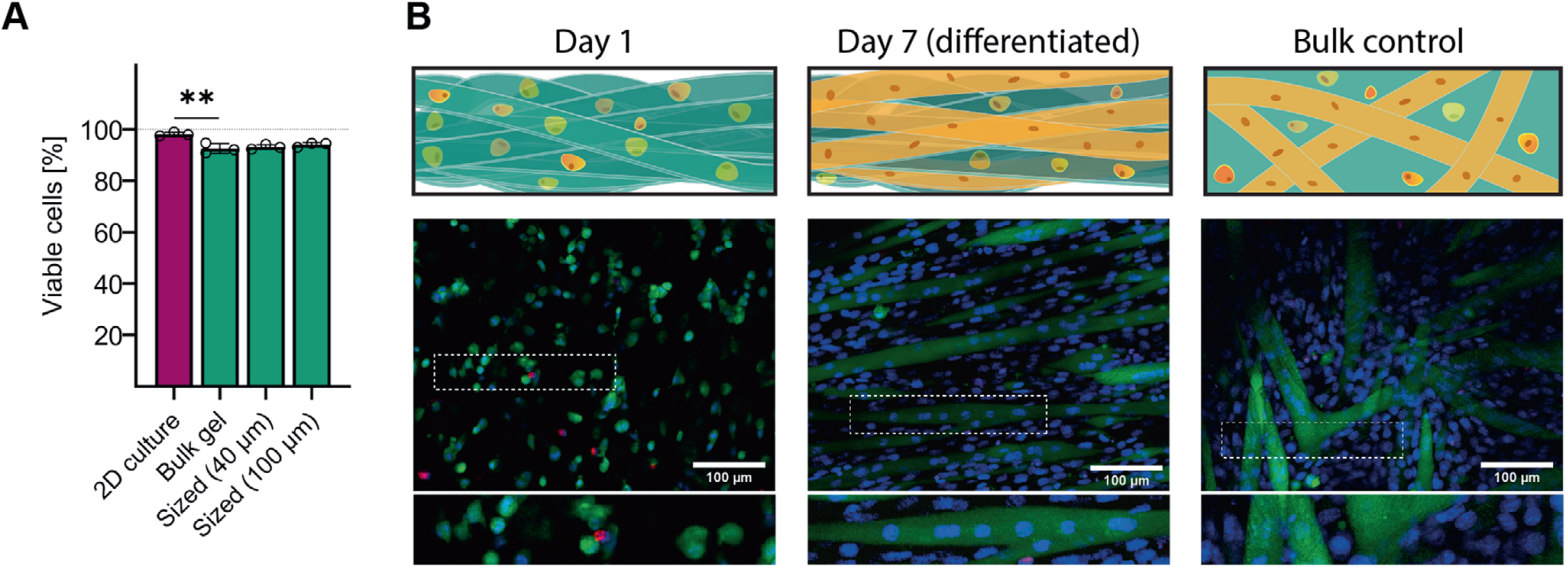
Microstrands can trigger aligned myotube formation: A) Myoblasts were embedded in bulk hydrogel with minimal reduction in cell viability and subsequently sized into cell‐laden microstrands without negative impact on viability. Data are presented as individual values as well as mean ± SD (*n* = 3). Viability of cells acquired from 2D culture was compared to those encapsulated in bulk gel with a two‐tailed *t*‐test (*p* < 0.01 indicated by **). Viability of cells encapsulated in bulk gel was compared to samples sized through meshes with a one‐way ANOVA (*p* = 0.588). B) Fluorescence images of cell‐laden entangled microstrands bioink (green = live, red = dead, blue = nucleus) show a healthy population of cells and quantification reveal a significant, but minor drop in viability when cells are encapsulated but not when sized through the grid. After differentiation, cell fusion and aligned myotube formation could be observed.

For anisotropic tissues like muscles, the Inside approach enables to confine cells within the gel microstrand and gives orientation during tissue maturation. Natural polymers such as collagen or its denatured form, gelatin, have been widely employed in the field of muscle tissue engineering.^[^ ^23^ ^]^ One important factor is the presence of RGD motives in gelatin which facilitates cell adhesion and has been shown to enhance myogenic differentiation.^[^ ^24^ ^]^ Therefore, gelatin was chosen as a hydrogel material and was combined with GelMA to avoid dissolution of the hydrogel during cell culture at 37 °C. As a first proof of principle, C2C12 cells were embedded within a bulk gel of 2% w/v gelatin and 2% w/v GelMA, and subsequently sized (cells inside gel phase). A significant, but minor drop of viability was observed for cells embedded in bulk hydrogel compared to freshly trypsinized cells (**Figure** **5**A, 2D 98.2 ± 0.6%; Bulk = 92.6 ± 1.6%; *n* = 6, *p* < 0.01). The viability of cells in hydrogels that have been sized into entangled microstrands was high (40 µm = 93.2 ± 0.8%, 100 µm = 94.1 ± 0.7%) and no significant difference in viability was found between either of the sized samples and the bulk gel controls (*n* = 3, *p* = 0.443). Differentiation media was added to trigger myotube formation of cultured C2C12 cells. Subsequent cytoplasmic Calcein AM staining combined with Hoechst 33342 revealed cell fusion and formation of multinuclear myotubes aligned along the direction of the microstrands (Figure 5B).

**Figure 6:**
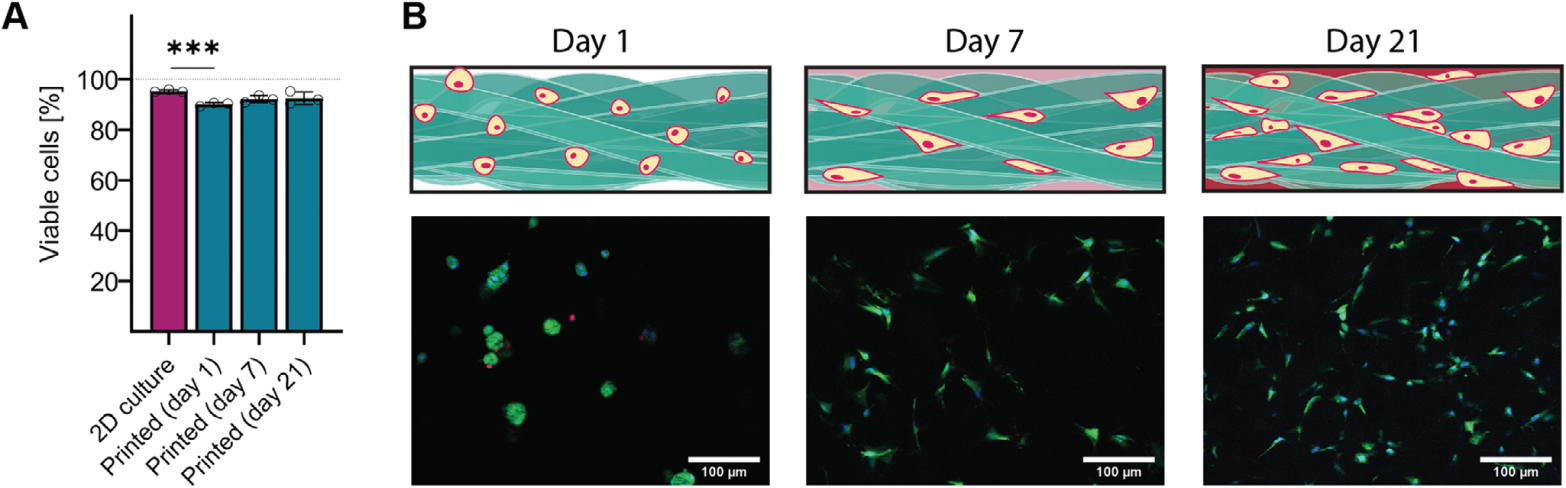
Entangled microstrands allow 3D bioprinting with high cell viability: A) Chondrocytes could be mixed with already prepared entangled microstrands (Outside) and 3D bioprinted with minimal impact on cell viability. Data are presented as individual values as well as mean ± SD (*n* = 3). Viability of cells acquired from 2D culture was compared to those inside a bioprinted construct with a two‐tailed *t*‐test (*p* < 0.001, indicated by ***). Differences of cell viability over time in bioprinted samples was compared with a one‐way ANOVA (*p* = 0.494). B) Fluorescence images showed high viability of cells after the printing process and cells adhering to the outer surface of entangled microstrands (green = live, red = dead, blue = nucleus). Chondrocytes occupied and proliferated in the void space between microstrands after 7 and 21 days of culture.

**Figure 7:**
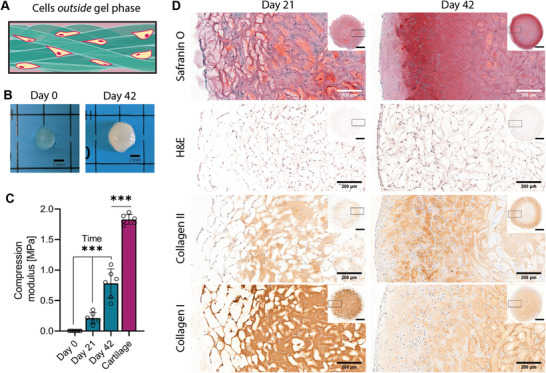
Entangled microstrands mature into cartilage‐like tissue: A) In this experiment, bovine chondrocytes were mixed with already prepared HA‐MA microstrands (Outside). B) Appearance of 3D bioprinted discs changed from translucent (day 0) to shiny‐white (day 42). C) Comparison of compression modulus of freshly bioprinted entangled microstrands, after in vitro culture and healthy articular cartilage. Data are presented as individual values as well as mean ± SD (*n* = 6). Development of compression modulus of bioprinted samples were compared over time with a Brown–Forsythe and Welch ANOVA (*p* < 0.001). Bioprinted samples cultured for 42 days were compared to bovine, native articular cartilage with a two‐tailed *t*‐test (*p* < 0.001). Asterisks indicate result of ANOVA for the factor time and result of *t*‐test. *** corresponds to *p* < 0.001. D) Histological staining to highlight the strong deposition of cartilaginous matrix within entangled microstrand scaffolds after 21 and 42 days of culture. Scalebar in overview picture represents 1 mm.

In the Outside approach, cells were present within the void fraction of the entangled microstrands and not embedded within the polymer network of the hydrogel itself. In this case, cells were less confined, since they were able to utilize the porous network to migrate, proliferate, and deposit extracellular matrix. The Outside approach was used to engineer de novo cartilage tissue. Hyaluronic acid derived hydrogels have been extensively used in cartilage tissue engineering, due to their biocompatibility and abundance in the extracellular matrix.^[^ ^25^ ^]^ Additionally, cells can interact with hyaluronic acid by CD44 surface receptors which was shown to influence synthesis of extracellular matrix and initiate chondrogenesis.^[^ ^26^ ^]^ Bulk HA‐MA hydrogel with medium crosslinking density was used since this condition had excellent rheological properties as well as print fidelity. Subsequently, sizing was conducted with meshes of 40 µm aperture to maximize void space in bioprinted constructs. Bovine chondrocytes were combined with HA‐MA microstrands and were 3D bioprinted into cylindrical discs. Cell viability was above 90% for the entire duration of the experiment (**Figure** **6**A, pre‐printing = 95.3 ± 0.5%; Day 1 = 90.1 ± 0.6%; Day 7 = 92.3 ± 1.1%, Day 21 = 92.6 ± 2%). Even though there was a significant drop in cell viability when bioprinted cells were compared to the original cell population (*n* = 3, *p* < 0.001), cell viability decreased by only 5.2 ± 1.2% (pre‐printing to Day 0). Cell viability remained high and no statistical difference in viability was found between printed cells at day 1, 7, and 21 (*n* = 3, *p* = 0.233). In the fluorescent Live/Dead staining, chondrocytes showed a rounded phenotype after 3D bioprinting, but proliferated and displayed a more elongated phenotype after 7 and 21 days in culture (Figure 6B). Immediately after fabrication, bioprinted scaffolds were translucent. After 42 days of culture, the discs appeared cartilage‐like and white, indicating deposition of a dense extracellular matrix (**Figure** **7**B). To confirm this, cultured tissue constructs were fixed and histologically stained for cartilage specific markers. A representative sample (*n* = 6) is depicted in Figure 7D. Staining with Safranin O showed an increased intensity with time, indicating strong proteoglycan content in the samples. Staining with hematoxylin & eosin (H&E) resulted in a contrast between stained cells and unstained entangled microstrands. While high levels of collagen type I were detected 21 days after fabrication, collagen type I staining was reduced after 42 days of maturation. Collagen type II was present after 21 days but restricted to the void space in between microstrands. After 42 days of culture, collagen II staining intensified and showed deposition inside the void space, as well as the hydrogel network of the entangled microstrands. The difference in staining between time‐points was particularly striking in the outer ≈400 µm of the sample. After 21 days, staining for cartilage‐ECM markers as well as abundance of cells in the outer area of the sample was lower compared to the central part of the scaffold. This distribution was reversed after 42 days of culture as there was a very dense deposition of ECM as well as cells in the outermost part of the scaffold. At the week 6 time‐point, cells in the outermost part did not show the initial pattern of thin lines and small clusters (also found in Live/Dead staining) anymore but had a more homogeneous distribution. This suggests that cells were able to migrate into the space previously occupied by the hydrogel microstrands.

The mechanical properties of cartilage tissue are highly important for its functions, especially the crucial ability to sustain load. Compression modulus of freshly bioprinted samples was very low (2.7 ± 0.3 kPa) but showed a significant increase over time (*n* = 6, *p* < 0.001), reaching 212 ± 83.7 kPa after 21 days and 780.2 ± 218.4 kPa after 42 days (Figure 7C). While this was still significantly lower when compared to native articular bovine cartilage which has a compression modulus of 1829.8 ± 72 kPa (*n* = 6, *p* < 0.001), this is a remarkable increase. Since the material used in this study had very little resistance to compression on its own, change in compression modulus could exclusively be attributed to the abundant deposition and maturation of extracellular matrix.

## 3 Conclusion

Advanced bioinks that excel at both biofabrication and cell compatibility are needed to address the challenges in the field of 3D bioprinting.^[^ ^8^ ^]^ Prior studies and recent reviews have documented the strength and potential of microgels for tissue engineering.^[^ ^2^, ^13^, ^27^ ^]^ However, production of microgels with conventional methods is challenging and limited by the materials which can be used. In this study, we demonstrated a simple and effective approach to deconstruct bulk gels into entangled microstrands which allows preparation of microgels in large quantities from many types of hydrogels. The facile preparation method allows the translation to most hydrogels used in tissue engineering.

Entangled microstrands can be 3D printed into constructs, closely mirroring the desired printing path. Since microstrands are solid elements, flow of microstrands after deposition on the buildplate is minimal. This can greatly increase resolution of printed constructs, especially with regards to multilayer constructs or sharp edges, in which cohesive forces cause layers of liquid bioinks flow into each other. Additionally, first demonstration of extrusion‐driven alignment of entangled microstrands was shown. These aligned hydrogel strands were effective cues which allowed embedded C2C12 cells to form oriented myotubes. Creation of relevant alignment in tissue engineering is critical for the creation of anisotropic structures like muscles, tendons, and nerves. Realization with spherical microgels, however, is extremely challenging, as this requires manipulation of the arrangements of microgels at a level, which has not been demonstrated yet. Further studies should therefore explore the full extent to which microstrands can align cells or multicellular constructs. In this study, C2C12 cells were embedded within the microstrands, but orientation of myotubes using the Outside approach is also possible, similar to how aligned myotubes form on micropatterned grooves.^[^ ^28^ ^]^

First results of entangled microstrands as a platform for tissue engineering have been promising. Two distinct ways to prepare cell‐laden bioinks were demonstrated: inside the gel phase and within the porous network outside the gel phase. Both methods yielded a remarkable percentage of viable cells for a microextrusion‐based approach, especially when compared to other extrusion based biofabrication methods.^[^ ^29^ ^]^ This is also true when directly compared to alternative microgel techniques, in which cells are encapsulated within hydrogel microbeads. Although cells are protected from shear stress in such a setup and no drop in viability is observed after 3D bioprinting, microfluidic preparation impairs viability at similar or greater levels and usually results in reported viabilities of 70–90%.^[^ ^11^ ^]^

Cell‐laden microstrands were also able to mature towards functional tissue. In an approach to create cartilage tissue, chondrocytes were incorporated in the void space between entangled microstrands, which they utilized to proliferate and deposit extracellular matrix. The strong staining of collagen type I after 21 days that reduced in intensity after 42 days can probably be attributed to initial dedifferentiation or not yet complete re‐differentiation of chondrocytes after 2D culture.^[^ ^30^ ^]^ Still, the abundant cartilaginous extracellular matrix deposited translated into a significant increase in stiffness, approaching that of native articular cartilage.

Even though successful maturation of tissues was shown, some aspects of this approach still offer room for improvements. The void fraction of the entangled microstrands was significantly lower compared to scaffolds based on spherical microgels.^[^ ^31^ ^]^ Our data shows that an interconnected network was formed that cells could utilize, still some tissues might benefit from an increased void fraction. For such tissues, tuning of the pores would be possible. Indeed, grids used in this study could be substituted by photo‐etched metal plates with apertures of custom cross section, for example, star shaped, to further increase distance between microgels or modulate the 3D environment. For spherical microgel systems, first attempts to characterize the influence of macroporosity and stiffness on cellular parameters such as viability, migration, and spreading have been undertaken.^[^ ^13^ ^]^ In future studies it will be interesting to see if these results can be reproduced with entangled microstrands or if the elongated nature, which will probably result in a more irregular void space provides different cell instructive properties.

Custom apertures could also help in further characterization of individual microstrands. In the presented system, irreversible entanglement of individual microstrands occurred during the production process. Due to the soft nature of these hydrogels it was not possible to “de‐tangle” or isolate individual strands without damage. Finding a way to study individual microstrands and characterize the aspect ratio would allow us to study how rheological properties, as well as scaffold stability and stiffness and cell migration are influenced by microstrand aspect ratio.^[^ ^17^ ^]^

Further limitations of the method are that tough and/or fibrillar hydrogels like externally crosslinked alginate were difficult to size. Hydrogels whose breaking strength is higher than their water retention capability, are squeezed rather than cut into microgels.

Entangled microstrands, like microbeads, are compatible with a multimaterial approach. Two paths forward to introduce heterogeneity into the method are envisioned, either using a starting bulk gel based on multiple polymers (either homogeneously mixed or compartmentalized within the bulk) or by mixing microstrands made from different bulk gels. The first would allow double network microstrands and double polymer systems in which only one sacrificial polymer (e.g., gelatin) is crosslinked and sized, while the other could be crosslinked at a later stage. In the second option, microstrands with differing mechanical properties or biological cargo could be combined to create precisely tuned bioinks for specific applications. This would also allow the creation of sacrificial microstrands to counter the limited void fraction compared to spherical microgel systems. Even though a homogeneous mixture of different microstrands without any damage to the material might be challenging to prepare, multi‐barrel syringes with intricate outlets could solve this problem. Such system would greatly benefit from an in‐line preparation, in which a bulk gel is automatically cut into microstrands and directly fed into an extrusion device. This would open the possibilities for an interchangeable printhead, compatible with current 3D bioprinters to further increase the ease of usability.

It should be acknowledged that this work is a first exploration of the potential of entangled microstrands. Further research and analysis is required to develop this approach and resolve the problems and challenges faced in the development of advanced bioinks. The simplicity and universality of this method though, provides an invaluable tool to the scientific community to create microstrand bioinks with unlimited material versatility from which novel architected hydrogels are possible.

## 4 Experimental Section

All chemicals were purchased from Sigma‐Aldrich unless stated otherwise.

##### Hyaluronic Acid Methacrylol

Hyaluronic acid with an average molecular weight of 1.7 MDa (1 g, HTL Biotechnology) was dissolved in ultrapure water (400 mL) and kept at 4 °C overnight to ensure complete dissolution. Ice‐cold DMF (267 mL) was added under continuous stirring. To start the reaction methacrylic anhydride (2370 µL) was added and the pH kept between 8–9 through the addition of 10 m NaOH for 4 h. Solid sodium chloride was dissolved in the solution to achieve a concentration of 0.5 m and the polymer was subsequently precipitated with ethanol (Merck). The precipitate was washed with ethanol, dried, and dissolved in ultrapure water. Solution was purified by diafiltration (Äkta 3, 10 NMWC hollow fiber). The purified product, hyaluronic acid methacrylate (HA‐MA) was lyophilized dissolved in deuterium (Cambridge Isotope Laboratories) and characterized by ^1^H NMR spectroscopy and stored at −20 °C in the dark until used.

NMR spectra were recorded at room temperature on a Bruker AV‐NEO 600 MHz spectrometer equipped with a TCI cryo probe. Spectra were obtained with 1024 scans using a 5 s recycle delay. To determine the degree of substitution, the ratio of the sum of the integrated peaks of the methacrylate protons (peaks at ≈6.1 and ≈5.6) and the integrated peak of the methyl protons of HA (≈1.9 ppm) was compared.

For gel preparation, HA‐MA was dispersed in PBS and kept at 4 °C until complete dissolution. HA‐MA solution was mixed with a 1% w/v lithium phenyl‐2,4,6‐trimethylbenzoylphosphinate (LAP) stock solution to create a 2% w/v HA‐MA and 0.05% w/v LAP solution and crosslinked by controlled photoexposure in the UV‐A range (Omnicure Series 100, 400 nm wavelength, 9.55 mW cm^−2^).

##### Gelatin Methacrylol

Gelatin type A (from porcine skin, gel strength ≈300 g Bloom) was dissolved in PBS at pH 7.4 and warmed up to 50 °C under vigorous stirring. Total used MA volume was split into five and after every addition, pH was adjusted with NaOH and the solution left to react for 30 min. After the last addition, reaction was diluted twofold and left to react for another 30 min. Product was cleaned by subsequent dialysis (10–12 kDa cutoff) against ultrapure water for 4 days. Solution was filtered, lyophilized, and stored at −20 °C until use. Modification of gelatin was quantified with ^1^H NMR spectroscopy (Bruker AV III HD, 400 MHz) with 2048 scans using D_2_O as solvent. To determine the degree of substitution, the lysine integration signal (2.95–3.05 ppm) of GelMA versus the lysine integration signal (2.95–3.05 ppm) of unmodified gelatin was compared. Phenylalanine signal at 7.2–7.5 ppm was used as internal reference. Degree of substitution was found to be 92%.

For gel preparation, GelMA and gelatin were dissolved in 70 °C hot PBS. GelMA solution was mixed with LAP stock solution (1% w/v) to achieve a final concentration of 2% w/v GelMA, 2% w/v gelatin, and 0.05% w/v LAP. Bulk gel was formed through thermoreversible gelation and sized into microstrands. To avoid melting of the gelatin/GelMA hydrogel microstrands during cell culture at 37 °C, additional crosslinking by controlled photoexposure in the UV‐A range was employed.

##### Hyaluronic Acid Transglutaminase

For HA‐TG hydrogel precursors, two different batches of HA were substituted with reactive glutamine (HA‐TG/Gln) and lysine (HA‐TG/Lys) residues, respectively following published protocols.^[^ ^21c^ ^]^

For gel preparation, HA‐TG/Lys and HA‐TG/Gln were dissolved in TBS buffer (150 × 10^−3^ m NaCl, 40 × 10^−3^ m CaCl2, 50 × 10^−3^ m TRIS, pH 7.6) and combined at equal volume to form HA‐TG solution. To initiate gelation, a solution of thrombin (Baxter, 500 U mL^−1^) and factor XIII (Fibrogammin, CSL Behring, 200 U mL^−1^) was added to form a gel with final concentrations of 3% w/v HA‐TG.

##### Iota‐Carrageenan

300 mg of iota‐carrageenan particles (Genuvisco CG‐131, GP Kelco) were added to 4 °C cold buffer solution (10 mL, 150 × 10^−3^ m KCl, 20 × 10^−3^ m HEPES, pH 7.4) to allow hydration of particles. Dispersion was then heated to 80 °C, stirred until complete dissolution, and transferred into a 10 mL syringe. Solution was cooled down and stored at 4 °C to form a 3% w/v gel.

##### Gelatin

Gelatin particles from porcine skin (type A, 300 mg) were added to 4 °C cold PBS (10 mL) and left to hydrate for 15 min with subsequent heating to 70 °C until complete dissolution. The solution was transferred into a 10 mL syringe and cooled down to 4 °C to form a 3% w/v bulk gel. To ensure reproducible results, gelatin solution was stored at 4 °C for 24 h to minimize variances due to the hardening of gelatin gels.

##### Hyaluronic Acid Divinyl Sulfone

A solution of 3% w/v hyaluronic acid, 3% w/v NaCl, and 0.2 m NaOH was prepared and stirred vigorously until complete dissolution of hyaluronic acid. Double the amount of divinylsulfone was added to the hyaluronic acid (w/w). Solution was mixed to ensure a homogeneous distribution and left to gel for 3 h at room temperature. Gel was then washed for 2 days in deionized water and used for experiments.

##### Carbodiimide Crosslinked Collagen (Collagen‐EDC)

Two different concentrations of Type I collagen solution (5 mg mL^−1^, Symatese; 80 mg mL^−1^, 3dbio) were mixed on ice to achieve a final concentration of 20 mg mL^−1^. An equal amount (w/w) of 3,3′‐dithiobis(propionohydrazide) (DTPHY) was directly dissolved in this solution and six times excess of 1‐ethyl‐3‐(3‐dimethylaminopropyl)carbodiimide (EDC) was first dissolved in MES buffer and subsequently added to the solution. Solution was mixed and left to react at 4 °C overnight to form a stable gel.

##### Deconstruction of Bulk Gels into Microstrands (Sizing)

To create entangled microstrands, bulk hydrogels were made inside a 10 mL syringe and manually pressed through a nylon sieve (Millipore, Filter code: NY41 for 40 µm, NY1H for 100 µm) and directly used for experiments.

##### Rheology

To assess rheological properties of the samples, all measurements were conducted on an Anton Paar MCT 301 rheometer equipped with a 20 mm parallel plate geometry at 25 °C in a humid atmosphere with a gap distance of 1 mm. Rheological properties of samples were examined by oscillatory shear sweeps (1% strain, 1–100 Hz), ramped shear rate (0.01–50 s^−1^), and strain sweeps (1 Hz, 0.01–1% strain) to evaluate storage and loss modulus, yield point, and shear thinning behavior. To investigate shear recovery properties, samples were exposed to repeating cycles of alternating phases of strain (1 Hz, 1% and 500% strain).

##### Compression Modulus Measurements

Disc shaped test specimen with 8 mm diameter and 2 mm height were stamped out of the bulk gel for acellular samples. For cellular samples, bioprinted constructs were used and exact dimension was measured before testing. Samples were tested by unconfined compression using a texture analyzer (TA.XTplus, Stable Microsystems). A 500 g load cell and a flat plate probe with a diameter of 15 mm were used. Samples were compressed to a final strain of 15% at a rate of 0.01 mm s^−1^. The compression modulus was calculated from the slope of the linear first 3% of the stress–strain curve.

##### Elastic Modulus

Bulk gels with defined crosslinking were prepared and dumbbell shaped specimens according to ISO 527‐2‐5B were stamped out. Specimen were attached in a custom clamp system and elongated until failure at a rate of 0.01 mm s^−1^.

##### Swelling

Bulk hydrogel discs were casted and weighed before immersion in PBS to induce swelling. For each time point, samples were removed from the PBS, blotted with a tissue to remove excess PBS, and weighed. The degree of swelling was calculated using following equation.

(1) $$\begin{array}{ccc} & & \mathrm{Degree}\;\mathrm{of}\;\mathrm{swelling}\left[\%\right]\\ & & \;=\frac{\left(\mathrm{mass}\;\mathrm{of}\;\mathrm{swollen}\;\mathrm{gel}\right)-\left(\mathrm{mass}\;\mathrm{of}\;\mathrm{non}-\mathrm{swollen}\;\mathrm{gel}\right)}{\left(\mathrm{mass}\;\mathrm{of}\;\mathrm{non}-\mathrm{swollen}\;\mathrm{gel}\right)}\times 100 \end{array}$$

##### Printing

Entangled microstrands were loaded into printing cartridges (Nordson EFD) and printed through a 410 µm conical needle (Nordson EFD) with a pneumatic driven extrusion 3D bioprinter (3D Discovery, RegenHU). 3D models for grid and disc structures were created with OpenSCAD version 2015.03‐2. 3D models were processed with Slic3r version 1.3.0 dev to create machine code (G‐Code).

##### Scanning Electron Microscopy

Entangled microstrands were extruded as straight lines through a 410 µm conical needle and collected on a glass plate. Bulk HA‐MA hydrogel and freshly made entangled microstrands were used as control. All samples were frozen in liquid nitrogen and lyophilized. For SEM analyses, the lyophilized samples were coated using Pt/Pd (80/20) at a thickness of 10 nm by a sputter coater (CCU‐010 HV, Safematic). The imaging was performed using a SEM instrument (JSM‐7100, JEOL).

##### Macroporosity

Entangled inks were made as described and submerged in PBS containing a high‐molecular‐weight, fluorescent FITC‐dextran (average molecular weight of 500 kDa). Entangled microstrands were then imaged by two‐photon microscopy (SP8, Leica).

##### Stability

Entangled microstrands were created as described, cut into cylinders, and transferred into well plates. Samples were submerged into PBS for up to 7 days and PBS was removed and exchanged after 5 min, 1 h, 24 h, and 7 days.

##### Cell Laden Microstrands (Inside)

C2C12 mouse immortalized myoblasts were obtained from ATCC. Cells were cultured in a humidified atmosphere (5% CO_2_, 37 °C) in Dulbecco's modified Eagle medium (DMEM GlutaMAX, Gibco) with fetal bovine serum (FBS, 10% v/v, Gibco) and gentamycin sulfate (10 µg mL^−1^, Gibco). Cells were passaged at 90% confluence and detached by Trypsin/EDTA (0.25%, Gibco).

Encapsulation solution (2% w/v gelatin, 2% w/v GelMA, and 0.05% w/v LAP) was prepared and kept at 37 °C to avoid solidification. Freshly detached C2C12 were added to the solution and gently mixed by continuous pipetting to achieve a final concentration of 10 × 10^6^ cells mL^−1^. After homogeneous distribution was achieved, solution was transferred into a syringe and cooled in an ice bath for 1 h, while the syringe was constantly rotated for the first 5 min to avoid sedimentation. After gelation period, cell containing bulk gel was pressed through a nylon grid (sized), secondarily crosslinked by photoexposure in the UV‐A range and kept in culture media. Cells were then cultured in differentiation medium composed of high glucose DMEM, insulin (1% v/v), transferrin and selenium mix (ITS+, Corning) and horse serum (2% v/v, Gibco), and gentamycin sulfate (10 µm mL^−1^). Medium was changed thrice a week.

##### Bioprinting of Entangled Microstrands (Outside)

Primary articular chondrocytes were isolated from the femoral cartilage of 6 month old calves obtained from the local slaughterhouse. Cartilage from the medial and lateral condyle was harvested, minced, and digested by collagenase solution (0.1% w/v, from *Clostridium histolyticum*) overnight. Cells were cultured in a humidified atmosphere (5% CO_2_ at 37 °C) and high‐glucose Dulbecco's modified Eagle's medium (DMEM, Gibco) supplemented with FBS (10% v/v), l‐ascorbic acid 2‐phosphate sesquimagnesium salt hydrate (50 µg mL^−1^), and gentamycin sulfate (10 µg mL^−1^). Cells were passaged at 90% confluence by detachment with trypsin/EDTA (0.25%) and used for experiments at passage 3.

To make bioink, entangled microstrands (HA‐MA, Med, 40 µm) and dense solution of bovine chondrocytes (100 × 10^6^ cells mL^−1^) were loaded in separate chambers of a double barrel syringe with a chamber ratio of 10:1 (Medmix). The two components were mixed by extrusion through a static mixing element (Medmix) to create cell‐laden entangled microstrands. Bioink was transferred into a printing cartridge (Nordson EFD) and printed into discs (*d* = 5 mm, *h* = 2 mm). To ensure long‐term shape fidelity, microstrands were annealed by UV‐A exposure (15 s). Constructs were cultured in high‐glucose DMEM supplemented with ITS liquid media supplement (1% v/v, Fisher Scientific), proline (40 µg mL^−1^), ascorbic acid (50 µg mL^−1^), gentamycin sulfate (10 µg mL^−1^), and TGF‐*β*3 (10 ng mL^−1^, Preprotech) for up to 6 weeks with full media change three times a week.

##### Live/Dead Staining

Bioprinted constructs were cut in half, washed with phenol‐free DMEM (Gibco), and stained with propidium iodide (0.5 µg mL^−1^), calcein AM (0.008 × 10^−3^ m), and Hoechst 33342 (5 µg mL^−1^) for 20 min and imaged with fluorescent light microscopy (ZEIS, Axio Observer Z1). Z‐stack images spanning 100 µm were acquired from the center of the scaffold and analyzed with FIJI. Experiment was done in triplicates, with the viability of each sample averaged over three pictures of randomly chosen positions inside the center of the hydrogel.

##### Histological Evaluation

Samples for histology were fixed in paraformaldehyde for 2 h, dehydrated, and paraffinized (LogosJ, Milestone). Paraffin blocks were cut with a microtome in 5 µm thick sections, dried deparaffinized, and hydrated. Tissue sections were stained with SafraninO, hematoxylin and eosin (H&E), Picosirius red, and Alizarin Red according to standard protocols.

For colorimetric, immunohistochemical stainings of collagen type I and II, sections were first digested in a hyaluronidase solution (1200 U mL^−1^, from *Streptococcus equi*) for 30 min at 37 °C. Sections were then washed and blocked with normal goat serum (NGS, 5% v/v) in PBS for 1 h at room temperature. Subsequently, slides were blotted and primary antibody in NGS (1% v/v) was added and left overnight in humidified atmosphere to avoid drying. Anti‐collagen type I antibody (mouse, Abcam #ab6308) was used at 1:1500 dilution, while anti‐collagen type II antibody (mouse, DSHB #II‐II6B3) was used at 1:200 dilution. On the following day, sections were washed with PBS and treated with 0.3% v/v H_2_O_2_ to quench any endogenous peroxidase or pseudoperoxidase activity to prevent nonspecific signals. After an additional washing step, secondary antibody (goat, anti‐mouse IgG (HRP), Abcam #ab6789) in NGS (1% v/v) solution was added and left under humidified atmosphere for 1 h. Secondary antibody was removed by three washes in PBS and DAB substrate (ab64238, Abcam) was added and left to react for precisely 3 min. Sections were washed again and counterstained by Mayer's hematoxylin solution. All samples were mounted and coverslipped with resinous mounting media (Eukitt) before imaging with a Pannoramic 250 histology slide scanner from 3D Histech.

##### Statistical Analysis

Statistical analysis was conducted with GraphPad Prism (v. 8.2.0 (425)). Alpha was set to 0.05 and statistical significance was assumed for *p* < 0.05. For statistical analysis data were used as acquired without pre‐processing. Throughout the manuscript, data are presented as individual values denoted by symbols as well as mean ± SD.

For acellular samples, storage modulus, elongation, and compression between samples were compared with one‐way analysis of variance (ANOVA) with a Tukey post hoc test (storage modulus: *n* = 9, elongation: *n* = 3, compression: *n* = 5). For swelling of samples, a mixed‐effects model with Geisser Greenhouse correction was used. Initial sample size was six samples per condition. Samples damaged over the course of the experiment were excluded after damage occurred. A minimum of three samples per condition were measured at all timepoints.

The influence of mesh size and crosslinking degree on the formation of macroporosity was investigated with a two‐way ANOVA. Sample sizes varied between conditions and a minimum of three samples were measured for each condition. To analyze the viability of encapsulated and bioprinted cells, one‐way ANOVA was used (*n* = 3). To compare viability of freshly trypsinized cells with encapsulated ones, an unpaired, two‐tailed *t*‐test was conducted (*n* = 3).

Mechanical properties of cellular, tissue engineered constructs were compared with a Brown–Forsythe and Welch ANOVA test due to unequal standard deviations (*n* = 6). Additionally, Day 42 samples were compared to native cartilage with an unpaired, two tailed *t*‐test (*n* = 6).

## Conflict of Interest

Tissue Engineering and Biofabrication lab at ETH Zurich has filed for patent protection on the technology described herein and M.Z. and B.K. are named as inventors on the patent.

## Author Contributions

B.K., M.L., and M.Z. designed the research. B.K. and M.Z. analyzed the data and wrote the paper. B.K., A.B., M.L., Y.T., and E.T. performed the research.

## Supporting information

Supporting Information

Supplemental Movie 1
