# Supplementary material for: 3D Bioprinting of Macroporous Materials Based on Entangled Hydrogel Microstrands
Source: Adv Sci (Weinh). 2020 Jul 19;7(18):2001419. doi: 10.1002/advs.202001419 (PMC7509724; doi:10.1002/advs.202001419)
Supplement: Supplementary file 1 — Supporting Information [file ADVS-7-2001419-s001.pdf]

Copyright WILEY-VCH Verlag GmbH & Co. KGaA, 69469 Weinheim, Germany, 2020.

Supporting Information

3D bioprinting of macroporous materials based on entangled hydrogel microstrands

*Benjamin Kessel, Mihyun Lee, Angela Bonato, Yann Tinguely, Enrico Tosoratti, Marcy Zenobi-Wong**

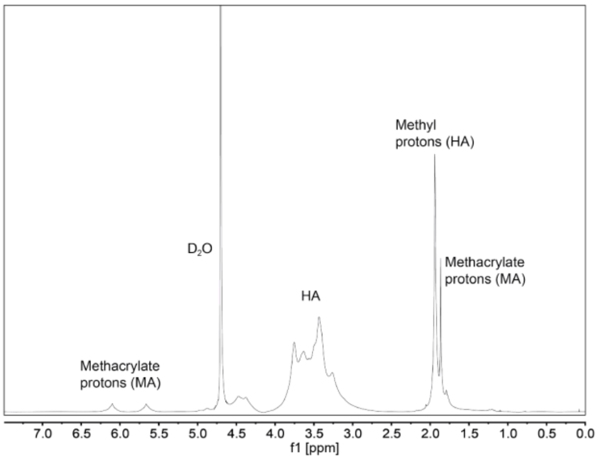

Figure S1. NMR spectra of synthesized HA-MA.

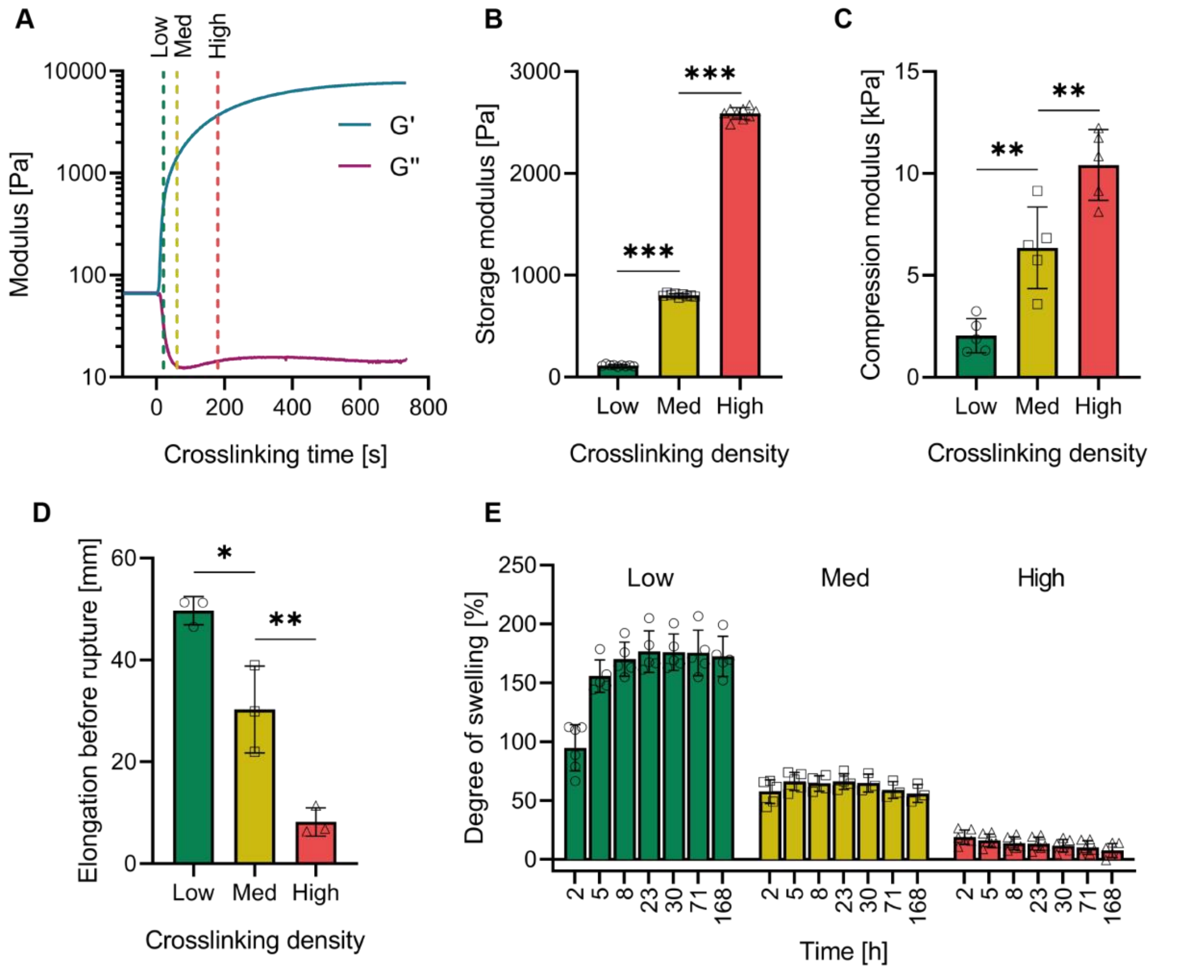

Figure S2. Mechanical properties of HA-MA gels: (**A**) Photocrosslinking behavior of HA-MA with three different time points representing different degrees of crosslinking (*Low*, *Med*, *High*). UV-mediated crosslinking is initiated at timepoint 0. (**B**) Storage modulus of bulk gels significantly differs between samples of varying crosslinking density ((n = 9, $P < 0.001$). (**C**) Compression modulus is significantly affected by crosslinking density (n = 5, $P < 0.001$). (**D**) Maxiumum elongation before rupture of dumbbell shaped sample happens after significantly different elongation (n = 3, $P < 0.001$). (**E**) Swelling ratio is different between samples made from different crosslinking degrees ($P < 0.001$). Damaged samples were excluded, a minimum of 3 samples were measured per timepoint and condition. For all plots, data is presented as individual values as well as mean ± SD. Overall significance was obtained from one-way-ANOVAs (B-D) and mixed-effects model (E). Asterisks in the figures indicate significant effects between groups acquired by Tukey post-hoc tests. *, **, *** indicate P-values of $P < 0.05$, $P < 0.01$, $P < 0.001$ respectively.

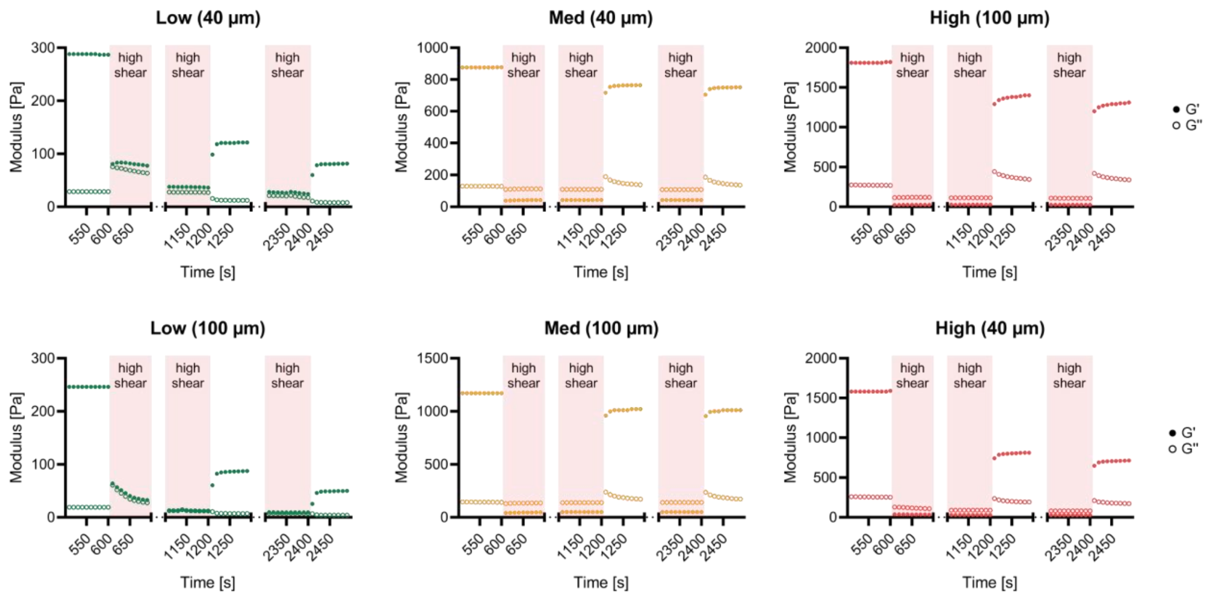

Figure S3. Individual shear recovery measurements of entangled microstrands presented in Figure 3E,F with a focus on the first two recovery events.

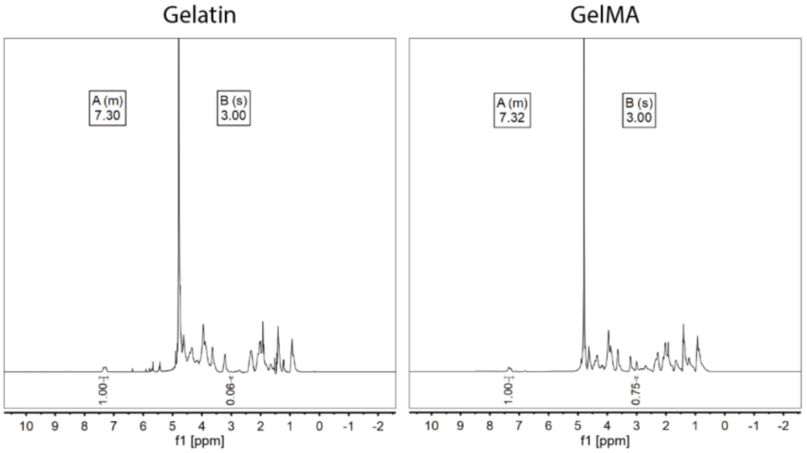

Figure S4. NMR spectra of unmodified gelatin and synthesized GelMA.

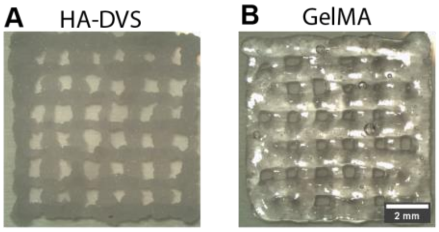

Figure S5. 3D printed GelMA and HA-DVS: Entangled microstrands prepared and printed from 3% (w/v) HA-DVS (**A**) and 2% (w/v) GelMA (**B**).

Table S1. Mechanical Properties of Different HA-MA Bulk Gels.

	Crosslinking time	Storage modulus	Compression modulus	Maximum elongation
Low	20 s	114.1±8.1 Pa	2.0±0.8 kPa	49.7±2.3 mm
Med	60 s	803.6±14.1 Pa	6.4±1.8 kPa	30.3±6.9 mm
High	180 s	2587.8±54.3 Pa	10.4±1.6 kPa	8.2±2.3 mm

Movie S1: Porosity of entangled microstrands.
